# Research Progress on Effects of Ginsenoside Rg2 and Rh1 on Nervous System and Related Mechanisms

**DOI:** 10.3390/molecules28237935

**Published:** 2023-12-04

**Authors:** Silu Liu, Weijia Chen, Yan Zhao, Ying Zong, Jianming Li, Zhongmei He

**Affiliations:** 1 College of Chinese Medicinal Materials, Jilin Agricultural University, Changchun 130118, China; liusilu0616@163.com (S.L.); chenweijia_jlau@163.com (W.C.); zhaoyan@jlau.edu.cn (Y.Z.); zongying7699@126.com (Y.Z.); lijianming4773@163.com (J.L.); 2 Key Laboratory of Animal Production, Product Quality and Security, Ministry of Education, Jilin Agricultural University, Changchun 130118, China

**Keywords:** ginsenoside Rg2, ginsenoside Rh1, nervous system, pharmacological effect, mechanism of action

## Abstract

Neurological-related disorders are diseases that affect the body’s neurons or peripheral nerve tissue, such as Parkinson’s disease (PD) and Alzheimer’s disease (AD). The development of neurological disorders can cause serious harm to the quality of life and functioning of the patient. The use of traditional therapeutic agents such as dopamine-promoting drugs, anticholinergic drugs, cholinesterase inhibitors, and NMDA receptor antagonists is often accompanied by a series of side effects such as drug resistance, cardiac arrhythmia, liver function abnormalities, and blurred vision. Therefore, there is an urgent need to find a therapeutic drug with a high safety profile and few side effects. Herbal medicines are rich in active ingredients that are natural macromolecules. Ginsenoside is the main active ingredient of ginseng, which has a variety of pharmacological effects and is considered to have potential value in the treatment of human diseases. Modern pharmacological studies have shown that ginsenosides Rg2 and Rh1 have strong pharmacological activities in the nervous system, with protective effects on nerve cells, improved resistance to neuronal injury, modulation of neural activity, resistance to cerebral ischemia/reperfusion injury, improvement of brain damage after eclampsia hemorrhage, improvement of memory and cognitive deficits, treatment of AD and vascular dementia, alleviation of anxiety, pain, and inhibition of ionic-like behavior. In this article, we searched the pharmacological research literature of Rg2 and Rh1 in the field of neurological diseases, summarized the latest research progress of the two ginsenosides, and reviewed the pharmacological effects and mechanisms of Rg2 and Rh1, which provided a new way of thinking for the research of the active ingredients in ginseng anti-neurological diseases and the development of new drugs.

## 1. Introduction

Ginseng is one of the main representatives of traditional Chinese medicine [1], as early as in the “Shen Nong Materia Medica” was recorded: “ginseng, taste sweet cold. The main tonic five viscera, peace of mind, settle the soul, stop shock and palpitation, remove evil spirits, bright eyes, happy, intelligence, long clothing light body health”. Ginseng is a rare traditional Chinese medicine, which has the function of prolonging life and invigorating vitality, and has been widely considered in clinical application [2].

**Figure 1:**
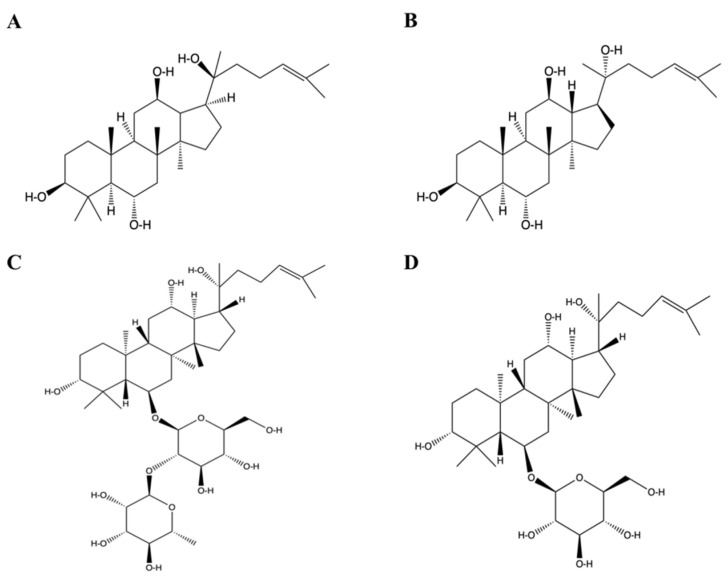
The structural formula of propanaxtriol saponins and ginsenosides Rg2 and Rh1. (**A**) 20 (S)-PPT. (**B**) 20 (R)-PPT. (**C**) Ginsenoside Rg2 structural formula. (**D**) Ginsenoside Rh1 structural formula.

Ginseng is rich in ginsenosides, polysaccharides, amino acids, and other components; ginsenosides are the main effective components [3]. Ginsenosides belong to tetracyclic triterpenoid saponins, which are composed of aglycones and glycogroups. After the glycogroups are degraded step by step, ginsenosides will be transformed into a series of secondary ginsenosides or saponins [4]. Their content is different in different plants of ginseng and different parts of ginseng, and it is different because of different extraction methods, cultivation years, and producing areas. Zang et al. [5] investigated the optimal process conditions for the preparation of 20(R)-Ginsenoside Rg2 and 20(R)-Ginsenoside Rh1 by acetic acid degradation and 20(R)-PPT by tartaric acid degradation using ginseng stem and leaf triolome saponins as raw materials by means of one-way as well as orthogonal tests. The results showed that the optimum degradation conditions for 20(R)-Ginsenoside Rg2 were as follows: material-liquid ratio of 1:20, degradation temperature of 80 °C, degradation time of 4 h, acetic acid content of 30%, and the conversion rate of the original ginsenotriol-type ginsenoside Re into 20(R)-Ginsenoside Rg2 was 24.29%. The conversion rate of 20(R)-Ginsenoside Rh1 into 20(R)-Ginsenoside Rh1 was 6.89% at 50% acetic acid, 1:20 ratio, 110 °C, 2 h degradation time. After degradation, ginsenriol-type saponins can be converted into secondary saponins and saponins with high biological activity and easy absorption, including proto-ginsenriol-type saponins 20(S/R)-PPT, 20(S/R) -ginsenosides Rg2 and 20(S/R) -ginsenosides Rh1. Their structures are shown in Figure 1. The common methods for procyantriol secondary saponins and aglycones are acid degradation, alkali degradation, enzyme degradation, and microbial degradation [6]. Current studies have found that primary ginsenoside secondary ginsenosides and saponins, such as ginsenoside Rg2 and ginsenoside Rh1, have better biological activity than common ginsenosides Re and ginsenoside Rg1 that have not been degraded, but the content of these secondary ginsenosides and saponins in original plants is minimal [7].

Ginsenosides Re, Rg1, and Rg2 are triol ginsenosides, which can be degraded into ginsenoside Rh1 by biodegradation technology. Modern pharmacological studies have shown that the triol ginsenosides Rg2 and Rh1 have strong pharmacological activities in the nervous system [8]; however, research reports in related fields are still relatively few.

Behavioral changes in the contemporary world, coupled with advances in medicine, have led to global demographic changes, particularly population ageing. The number of people over 60 is growing at the highest rate of any other age group, and the population in the coming decades is expected to double its current number by 2050 [9]. The aging population is also prone to homeostasis [10], metabolic disorders [11], cardiovascular diseases [12], neurodegenerative diseases [13], and even cancer [14]. Global prevalence data show that neurodegenerative diseases are one of the main causes of ageing populations [15]. In order to better research the development and application of ginseng in nervous system-related diseases, this paper reviews the effects of ginsenosides Rg2 and Rh1 on the nervous system, aiming to provide new ideas for the development of new drugs for ginsenosides Rg2 and Rh1.

## 2. Effect of Ginsenoside Rg2 on Nervous System

### 2.1. Protective Effect on Nerve Cells

Studies have shown that Rg2 has a strong protective effect on nervous system damage caused by various causes, and plays an antioxidant and anti-apoptotic role [16]. Parkinson’s disease (PD) is a common neurological disorder characterized by tetanus, quiescent tremor, and slow movement [17]; its pathogenesis is related to the progressive loss of dopaminergic neurons in the substantia nigra and the depletion of dopamine in the striatum. Dopamine (DA) is the catecholamine neurotransmitter in the brain [18]; 6-Hydroxydopamine hydrobromide (6-OHDA) is the hydroxylated analogue of DA. Alterations in intracellular signaling pathways, including the MAPKs pathway, are associated with 6-OHDA toxicity, and extracellular signal-regulated protein kinase (ERK) activation and c-jun N-terminal kinase (JNK) activation have been observed in a variety of models [19,20].

Neuroblastoma (NB) is an embryonic malignant tumor that affects the normal development of the adrenal medulla and paravertebral sympathetic ganglion in early childhood [21]. This study confirmed that ginsenoside Rg2 significantly inhibited the toxicity of 6-OHDA on SH-SY5Y cells, promoted the growth of PC-12 cells, and reduced 6-OHDa-induced ERK phosphorylation [22].

NaLi [23] investigated the effect of ginsenoside Rg2 on glutamate-induced neurotoxicity in PC12 cells. The results showed that glutamate decreased the activity of PC12 cells, increased Ca^2+^, led to the overproduction of malondialdehyde (MDA) and nitric oxide (NO), and upregulated the protein expression levels of calpain II, caspase-3, and amyloid β-Protein (1-40) (Aβ1-40). The above trends were reversed after administration of Rg2, which significantly reduced glutamate-induced neurotoxicity. In conclusion, the mechanism of Rg2 may be the inhibition of glutamic acid (Glu) release to resist neurotoxicity. To observe the effect of ginsenoside Rg2 on ischemic reperfusion injured rat neurons, it was found that the expression of N-Methyl-D-aspartic acid (NMDA) receptor, amyloid Precursor Protein (APP), and Aβ1-40 decreased, the expression of HSP70 increased significantly, and the cell activity was significantly enhanced. The expression levels of NMDA receptor and APP decreased after administration of Rg2. The cytoplasmic expression of heat shock protein 70 (HSP70) decreased and the production of Aβ1-40 increased; cell activity increased [24], which has a protective effect on nerve cells.

The results showed that ginsenoside Rg2 had the best protective effect on PC12 cells induced by amyloid β-Protein (25-35) (Aβ25-35) at the dose of 5–20 μg/mL. Compared with the blank group, the cell viability of the ginsenoside Rg2 pre-protected group was significantly increased and showed strong concentration dependence. Meanwhile, the release of lactate dehydrogenase induced by Aβ25-35 was decreased, and the ratio of B-cell lymphoma-2 (Bcl-2)/Bcl-2-associated X (Bax) was upregulated. The mechanism may be upregulated Bcl-2 expression and downregulated Bax expression. The regulation of the Phosphatidylinositide 3-kinases (PI3K)/Protein kinase B (Akt) signaling pathway protects PC12 cells from Aβ25-35-induced apoptosis [25].

### 2.2. Treatment of Vascular Dementia

Vascular dementia (VD) is a group of mental and cognitive disorders caused by cerebrovascular diseases [26]; it is a chronic progressive disease, and the course of the disease is often manifested as a wave or step deterioration, and is often accompanied by cerebrovascular disease [27]. Studies have shown that ginsenoside Rg2 can increase the content of monoamine transmitters, so as to improve the excitability of the cerebral cortex, activate the learning and memory process after cerebral ischemia-reperfusion injury (CIRI), and improve the learning and memory ability of VD model rats [28].

Zhang Li et al. [29] found that ginsenoside Rg2 can significantly improve learning and memory impairment in rats, and its mechanism may be related to its effective regulation of the expression of glutamate receptor subunit genes in the brain. The effect of ginsenoside Rg2 on the nervous system performance and memory of VD rats may be related to anti-apoptosis. A VD rat model was established, neurological evaluation was performed 24 h after reperfusion, and Y maze memory performance was evaluated 48 h after reperfusion. The relationship between the expression of Bcl-2, BAX, HSP70, and recombinant Tumor Protein P53 (P53) proteins and cell apoptosis was detected by immunocytochemistry. The results showed that compared with the VD group, the ginsenoside Rg2-positive group significantly improved the neurological response and memory ability, and increased the expression of Bcl-2 and HSP70, but decreased the expression of Bax and p53. These findings suggest that Rg2’s ability to regulate the expression of apoptosis-related proteins may be a potential therapeutic strategy for VD or other ischemic injuries.

### 2.3. Anti-Ischemia Reperfusion Brain Injury

Ischemic cerebrovascular disease is a common disease. Restoring blood supply within a certain time after cerebral ischemia can aggravate the injury of ischemic cells and cause ischemia reperfusion injury [30,31].

Li Taidong et al. [32] discussed the intervention effect of ginsenoside Rg2 on cerebral ischemia-reperfusion injury in rats, and the results showed that ginsenoside Rg2 can reduce the expression of Aβ1-40, APP, and NMDA receptor protein (NR1) caused by cerebral ischemia-reperfusion injury, and improve learning and memory ability. It may also play a protective role against cognitive dysfunction; Chen Meihua [31] evaluated the neurological function of cerebral ischemia reperfusion rats by using the Longa5 scale. Ginsenoside Rg2 can reduce the cerebral infarction area and improve cholinergic nerve activity by reducing the neurological function score of cerebral ischemia reperfusion rats, so as to play a protective role in cerebral ischemia reperfusion injury.

The Oxygen glucose stripping/Reperfusion (OGD/R) cell model is an internationally recognized in vitro model commonly used to simulate CIRI [33]. It has been reported that pretreatment with ginsenoside Rg2 and its stereoisomers [20(R)-Rg2 and 20(S)-Rg2] can improve the activity of OGD/R model cells [34]. By analyzing the mechanism of action, ginsenoside Rg2 may play a protective role by improving the antioxidant and calcium overload ability of cells.

### 2.4. Anti-Anxiety, Anti-Pain Depression-like Behavior

Clinically, chronic pain patients are often accompanied by depression, and some depression patients also have chronic physical pain symptoms [35]. Therefore, the relationship between pain and depression has become a focus of recent research. Studies have shown that chronic pain can induce depression-related behaviors [36].

The study confirmed the ability of ginsenoside Rg2 to alleviate the chronic constriction injury (CCI) model in rats under the route of administration of intraperitoneal injection [37]. Mechanical foot-constriction reflex thresholds and thermal radiation foot-constriction latencies, pain sensitivity, anxiety, and depression-like behaviors were observed and noted in CCI rats, and also examined using the light and dark box experiments, shuttle time, and forced-swimming experiments; it was observed that the administration of ginsenoside Rg2 was able to inhibit mechanical and thermal pain sensitivity, and improve the depressive state of the rats with CCI.

Major depression is a common neuropsychiatric disorder [38]; this disease is characterized by a high lifetime prevalence and a high suicide rate [39].

Ying Ren et al. [40] evaluated the antidepressant effect of ginsenoside Rg2 in mice and identified the possible mechanism. Rg2 and fluoxetine (positive control, 20 mg/kg) at doses of 10 and 20 mg/kg had significant antidepressant-like effects in both the forced swimming test and the tail suspension test. Two weeks of administration of Rg2 and fluoxetine completely reversed depression-like symptoms caused by chronic mild stress in mice. In addition, western blotting showed that Rg2 significantly increased the brain-derived neurotrophic factor (BDNF) signaling pathway in the hippocampus, and that the use of Tyrosine Kinase receptor B short hairpin RNA (TrkB shRNA) completely blocked the antidepressant effect of Rg2 in mice. Thus, Rg2 exerts an antidepressant-like effect by promoting the BDNF signaling pathway in hippocampus. 

Post-traumatic stress disorder (PTSD) is a mental illness [41,42]. PTSD was associated with behavioral deficits in rats exposed to a single-long stress (SPS) induction [43,44]. It was confirmed that Rg2 doses of 10 and 20 mg/kg reversed the decrease in open arm time and entry times in the elevated plus maze test (EPMT) and the increase in freezing time in the situation-fear experiment, without affecting the autonomic activity of mice. Meanwhile, Rg2 blocked the decrease in progesterone, allopregnenolone, 5-hydroxytryptamine (5-HT), 5-hydroxyindoleacetic acid (5-HIAA), corticotropin releasing hormone (CRH), corticosterone (Cort), and adrenocorticotropic hormone (ACTH) in the brain and serum [45]. In summary, Rg2 alleviates behavioral deficits associated with PTSD through neurosteroid biosynthesis, normalization of the serotonergic system, and HPA axis dysfunction.

### 2.5. Anti-Alzheimer’s Disease

AD is a neurodegenerative disease with insidious onset and progressive development [46]; it is characterized by progressive memory impairment, aphasia, apraxia, agnosia, personality change, and executive dysfunction [47,48]; it has gradually become one of the most serious diseases endangering human health.

Ginsenoside Rg2 can improve the learning and memory ability of AD rats [49], prevent the formation of age spots [50], improve the degenerative changes in neurons in the cerebral cortex and hippocampus of AD rats, and play a role in the prevention and treatment of AD.

Shangchong et al. [51] revealed that ginsenoside Rg2 significantly enhanced the learning memory ability of scopolamine-induced AD mice, and the mechanism may be related to the inhibition of acetylcholinesterase (AChE) activity and the increase in choline acetyltransferase (ChAT) activity in the hippocampus and pre-cortex of mice. Thus, increased Acetylcholine (ACh) content is related.

Wu Cong and colleagues [52] used Aβ25-35 to establish an animal model of AD in order to investigate the effects of ginsenoside Rg2 on the structure of hippocampal neurons and synaptophysin (SY) expression in the rat hippocampus of the AD model group, and observed a significant reduction in the number of hippocampal neurons in the AD rats, light SY immunohistochemical staining, increased heterochromatin in the nucleus of neurons, swollen intracytoplasmic mitochondria, broken ridges, and the appearance of lipofuscous granules. Protrusions in the nerve felt were cavitated, and neurofilaments and mitochondria were reduced. The overall morphology and ultrastructure of hippocampal neurons improved to a certain extent after administration of ginsenoside Rg2. The deepening of SY immunohistochemical staining confirmed the protective effect of Rg2 on the structure of hippocampal neurons and SY expression in AD rats.

### 2.6. Protective Effect on Neuronal Injury

CuiJing et al. [25] established a rat model of Alzheimer’s disease (AD) by injecting Aβ25-35 (1 μg/μL) into rats. The results showed that the cognitive function of AD rats was significantly improved in the Rg2 group. After administration of ginsenoside Rg2, Aβ25-35-induced hippocampal CA1 tissue damage was inhibited, the Bcl-2/Bax ratio was upregulated, capase-3 division was weakened, and Akt phosphorylation was enhanced. These trends confirmed that ginsenoside Rg2 improves Aβ25-35-induced cognitive dysfunction by activating the phosphatidylinositol 3-kinase/protein kinase B (PI3K/Akt) signaling pathway.

Wang’s team [53] found that Rg2 has neuroprotective effects on hypoxia-induced hippocampal neuronal damage. Experiments using hypoxia-induced neonatal rats showed that administration of Rg2 alleviated hypoxia-induced neuronal apoptosis, attenuated intracellular Ca^2+^ overload, increased SOD activity, and reduced the levels of MDA and NO in the cell culture supernatant.

### 2.7. Improvement of Brain Injury after Bleeding in Eclampsia Models

Pre-eclampsia is a life-threatening pregnancy disorder unique to humans, and a major cause of maternal and neonatal morbidity and mortality. Women who survive pre-eclampsia have a reduced life expectancy and an increased risk of stroke, cardiovascular disease, and diabetes, while babies born with pre-eclampsia pregnancies are at increased risk of preterm birth, perinatal death, and neurodevelopmental disorders, and cardiovascular and metabolic disease later in life [54]. In recent years, there has been an increasing incidence of hemorrhagic strokes after birth among women who had pre-eclampsia during pregnancy [55,56].

The effect of ginsenoside Rg2 on pregnancy outcomes and brain injury after intracerebral hemorrhage (ICH) was evaluated in a rat model of pre-eclampsia. Pre-eclampsia was induced by N(ω)-nitro-L-arginine methyl ester in rats, and the ICH model was prepared by injection of bacterial collagenase into the striatum [57].

**Figure 2:**
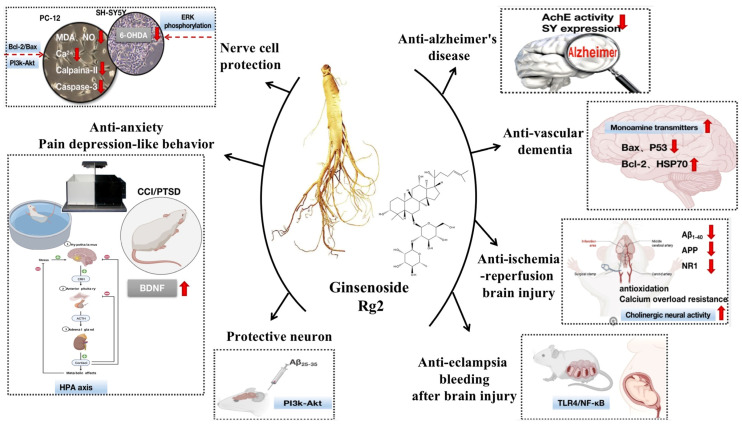
Ginsenoside Rg2 activity against neurosystem-related diseases.

Ginsenoside Rg2 significantly improved fetal survival, placenta, and body weight. The Garcia test score of ginsenoside Rg2 treatment rats was significantly improved compared with the pre-eclampsia group. The results showed that ginsenoside Rg2 improved pregnancy outcomes in a rat model of pre-eclampsia without reducing blood pressure and urine protein levels. Meanwhile, ginsenoside Rg2 improved ICH-induced neurological disease and blood-brain barrier dysfunction in animal models of pre-eclampsia by modulating the toll-like Receptor 4 (TLR4)/nuclear factor kappa-B (NF-κB) signaling pathway, the above contents are shown in Figure 2.

## 3. Effect of Ginsenoside Rh1 on Nervous System

It was found that after oral administration of ginseng extract, the proginsenotriol ginsenoside was mainly hydrolyzed or metabolized to Rh1 in the gastrointestinal tract [58]. In addition, Rh1 has been reported to effectively stimulate the central nervous system and enhance mental acuity and intellectual performance [59]. Multiple studies have shown that Rh1 has neuroprotective effects [60] and potential anti-tumor effects [61], and can be used as an adjunct therapy for chronic inflammatory diseases.

### 3.1. The Regulatory Effect on Neural Activity

Lee et al. [62] compared the effects of seven ginsenosides on neural activity by measuring changes in fEPSP slope. Rh1 was moderately effective (46.1 ± 6.7%) in inhibiting fEPSP at 100 µg/mL. Complete elimination of fEPSP was observed in the case of the non-NMDA receptor antagonist 6-cyano-7-nitroquinoxalin-2-3-dione, but not in the case of the NMDA receptor antagonist (2R) -amino-5-phosphonylvalanoic acid. This suggests that Rh1 may play a role in regulating synaptic activity mediated by non-NMDA receptors. Furthermore, a cell toxicity model was established for SH-SY5Y cells using 60 μM 6-OHDA, and significantly reduced toxicity was observed at doses of 10–20 µM Rh1, due to reduced ERK 1/2 phosphorylation after Rh1 treatment. Rh1 increased the percentage of PC-12 cells with neurites compared to the control group [22].

### 3.2. Improve Memory and Cognitive Impairment

Sleep deprivation (SD) is associated with impaired memory by inducing oxidative stress [63]. Cong et al. [64] were intraperitoneally administered ginsenoside Rh1 and Modafinil (0.42 g/kg) at doses of 20 μmol/kg and 40 μmol/kg. During the subsequent 14-day SD model, cognitive ability was assessed by behavioral tests, the cortex and hippocampus were dissected and homogenized, and oxidative stress levels were assessed. The results suggest that Rh1 can prevent SD-induced cognitive impairment, and the mechanism of action may be through the ability to reduce oxidative stress in the cortex and hippocampus. In passive avoidance experiments, Rh1 pretreatment at doses of 5 and 10 mg/kg significantly improved the learning and memory of scopolamine-induced memory impairment mice [65] or saline treatment mice [66]. The Morris water maze test showed that Rh1 (10 mg/kg) significantly shortened the escape latency, increased the number of crossings and platform time, and significantly improved the spatial learning ability.

### 3.3. Anti-Alzheimer’s Disease

AD is characterized by the accumulation of beta-amyloid plaques and highly phosphorylated tau proteins in the brain [67,68]. A large number of studies have confirmed that signaling pathways such as PI3K/Akt play a key role in regulating cell survival, movement, transcription, metabolism, and cell cycle progression [69]. Recent studies have shown that disruption of such signaling pathways leads to oxidative stress and cell death [70]. Eventually, it leads to the progression of neurodegenerative diseases. SH-SY5Y cells were found to be treated with beta-amyloid oligomeric alone or in combination with ginsenoside Rh1. Rh1 was observed to attenuate beta-amyloid-induced oxidative stress and cell death by activating the PI3K/Akt signaling pathway [69]. In conclusion, ginsenoside Rh1 may be an effective therapeutic agent for AD.

The effect of Rh1 at a medium dose (10 mg/kg) was comparable to that of huperzine A.

**Figure 3:**
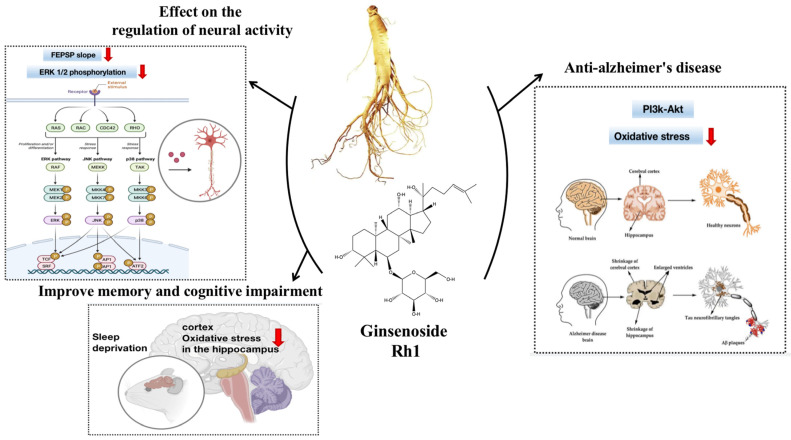
Ginsenoside Rh1 activity against neurosystem-related diseases.

Bi et al. [71] explored the activity of ginsenoside Rh1 using behavioral assays to detect cognitive abilities in mice with scopolamine-induced AD. Behavioral tests showed that mice given Rh1 had a significantly shorter avoidance latency, a reduced number of regression test errors, a shorter retention time and distance within the effective zone, a significant improvement in spatial discrimination behavioral deficits, and that Rh1 increased the content of Ach in the hippocampal region of the brain, the above contents are shown in Figure 3.

## 4. Discussion

Natural Chinese herbal medicines are highly safe and represent a gold mine for the development of new therapies for a wide range of major diseases [72,73]. According to the World Health Organization (WHO), about 4 billion people worldwide use Chinese herbs for disease treatment, and the development and utilization of Chinese herbs will be on the rise worldwide in the next 10 years.

Researchers have been studying the activity of natural ingredients in Chinese herbs in alleviating diseases and promoting human health [74]. Polysaccharides are the main immunologically active substances in Chinese herbs. Study has shown that dandelion polysaccharides can alleviate CCL4-induced pulmonary fibrosis in rats, and mitigate inflammatory responses and oxidative stress injury by scavenging free radicals and attenuating inflammatory cell activation [75]; Li et al. [76] used nimodipine as a positive control group and found that Panax notoginseng polysaccharides improved the antioxidant capacity of brain tissues and inhibited overproduction of inflammatory cytokines, and had a protective effect on rat I/R injury with protective effects. Saponins are widely distributed in nature, and it has been found that saponins extracted from traditional Chinese medicines have various pharmacological activities, such as anti-tumor [77], anti-inflammatory [78], anti-cancer [79], prevention and treatment of cardiovascular diseases [80], and that Ginsenoside Rg3 protects the myocardium through the Akt/eNOS signaling pathway and increases the Bcl-2/Bax ratio [81]. Flavonoids are secondary metabolites of plants, which are widely present in plants, and have antioxidant, anti-inflammatory, anti-tumor, and immune-enhancing effects. Hawthorn is a medicinal homoeopathic drug commonly used in the treatment of heart disease. Currently, in vivo and in vitro studies have demonstrated that hawthorn extracts have a wide range of cardiovascular pharmacological effects, including antioxidant, antiplatelet aggregation, endothelial protection, and anti-I/R injury; and crataegus extract improved the liver histological injury and decreased the contents of mucoglycan and protein in liver cells, alleviating the liver injury induced by CCl4 in rats [82]. *Ginkgo biloba* L. extract (GBE) is a commonly used herbal medicine for the prevention and treatment of myocardial ischemia, which plays an important role in cardioprotection through the comprehensive regulation of multiple metabolic pathways. GBE has a positive effect on cognitive and neurological function through vascular flow regulation and platelet activation antagonistic factors, thereby protecting the brain from ischemic damage [83].

Polyphenols have antioxidant and anti-atherosclerotic properties that inhibit peroxyl radical-induced DNA strand damage, inhibit platelet aggregation, reduce low-density lipoprotein (LDL) oxidation, and improve vascular endothelial function [84]. Resveratrol (RSV), a polyphenol compound found in grapes, red wine, mulberries, white flamingos (Veratrum grandiflorum O. Loes), and Polygonum cuspidatum, has been widely studied for its beneficial effects on human health, including neurological protective effects [85,86], cardioprotective [87], and anti-tumor [88]. Hibiscus sabdariffa L. (HS, Roselle) belongs to the Malvaceae family; the dried flowers of HS are rich in polyphenols and anthocyanin pigments, and have been reported to have anti-hyperlipidemia, antioxidant, anti-anemia, and anti-inflammatory effects. Alaaeldin Ahmed Hamza et al. [89] established a hepatotoxicity model using cisplatin and confirmed that Hibiscus sabdariffa alleviates liver damage by reducing oxidative stress and reducing apoptotic activity in the liver while enhancing its anti-cancer efficacy. Salidroside (Sal) is a phenylpropyl alkyl glycoside isolated from the plant Rhodiolarosea L. Studies have shown that Sal is able to exert neuroprotective effects on aging hippocampal neurons and naturally aging mice through the PI3K/Akt/TERT pathway [90].

With the aging of the global population, thousands of elderly people suffer from neurological disorders. The physical and mental health of the patients is also greatly affected. Most traditional drug compounds have strong side effects on central nervous system (CNS) diseases [91], and compared with traditional drugs, biomolecules in herbal medicines have high safety and sizable cost [92], and their application in the field of disease treatment is gradually expanding [93].

Ginseng, as a traditional Chinese medicine, has significant pharmacological activities in the treatment of neurological disorders caused by various reasons. Rg2 and Rh1 are important active components of ginsenosides present in C.A. ginseng mey, P. quinquefolium, and other ginsengs, which have also shown strong activity in the treatment of central nervous system diseases. By collecting, classifying, and summarizing recent research progress from 2023 to 2007 on the pharmacological activities of Rg2 and Rh1 in the treatment of neurological-related diseases, we found that they can modulate various signaling molecules and pathways in the nervous system and exert pharmacological activities in the nervous system.

Inflammatory diseases of the nervous system are very common. Inflammation can induce mitochondrial dysfunction and, conversely, inflammation can be caused by mitochondrial dysfunction [94]. Mitochondrial dysfunction and neuroinflammation may contribute to the development of neurodegeneration and neurodegenerative diseases [95]. Glutamate is a major excitatory neurotransmitter that acts at various excitatory synapses in the nervous system. Studies have confirmed that high concentrations of glutamate can induce neuronal damage and apoptosis [96]. The glutamate-induced Ca^2+^ overload hypothesis has been widely accepted as the mechanism of neuronal injury in glutamate-induced excitotoxicity [97].

Calcium overload is another important pathological mechanism associated with AD [98,99]. Ca^2+^ overload has been reported to cause a range of neuronal injuries, with intracellular Ca^2+^ overload triggering neuroinflammation, cytochrome C release, and apoptosis [100], and elevated intracellular free Ca^2+^ levels also mediating the activation of Ca^2+^-dependent enzymes. In addition, dysregulation of calcium ion homeostasis is an important factor in age-related neuronal damage, and even mild impairment of calcium ion signaling can induce deleterious changes, including neuronal death. Calcineurin (CN) has been reported to be a calcium-dependent protein phosphatase [101]. Nuclear factor activating T cells (NFAT) can be dephosphorylated to regulate its target genes. Calcineurin is mainly involved in calcitoxic neuronal damage and necrosis. It has been shown that Rg2 reduces intracellular Ca^2+^ levels and decreases lipid peroxidation in neuronal cells. In addition, caspase 3 is considered to be a key executor of apoptosis, and Rg2 can inhibit the expression of calpain II and caspase-3. These results suggest that ginsenoside Rg2 has anti-apoptotic properties.

VD is an age-related neurological disorder in which cognitive impairment is attributed to vascular lesions such as ischemic stroke, hemorrhagic stroke, cerebral ischemia, and hypoxia [102]. In China and other Asian countries, the disease is very common among the elderly and is the second most common subtype of dementia after AD. Patients with AD present with neurovascular abnormalities in the brain, and vascular lesions are often associated with some of the pathological features of AD, such as deposition of A-beta plaques and neurofibrillary tangles consisting of phosphorylated tau [103], implying that there is a relevance association between AD and VD. Based on imaging and pathologic changes, vascular dementia can be divided into several subtypes, including multi-infarct dementia, small-vessel dementia, strategic infarct dementia, hypoperfusion dementia, hemorrhagic dementia, hereditary vascular dementia, and cardiovascular disease AD.

The pathogenesis of VD is complex, but studies have confirmed that abnormalities of the glutamatergic system in the brain are closely related to the pathogenesis of VD [104]. In general, CIRI increases apoptosis, activates NMDA receptors, and leads to Ca^2+^ influx, resulting in intracellular calcium overload [105]. The survival of cortical neurons was significantly decreased after OGD/R. However, the decrease in survival was inhibited after administration of ginsenoside Rg2, suggesting a neuroprotective effect of ginsenoside Rg2 against this injury. Meanwhile, activation of caspase-3 promotes apoptosis in OGD/R conditions, while Rg2 effectively inhibits the upregulation of caspase-3.

NMDA receptors and α-AMPA receptors are the main pro-ionic glutamate receptors [106]. The subunits distributed in the hippocampus mainly include NR1, NR2A, NR2B, and GluR2, which play important roles in learning and memory processes in the hippocampus. The number and functional activity of glutamate receptors change during VD [107]. The mechanism by which Rg2 ameliorates learning and memory deficits in VD-like rats may be related to the glutamatergic system, which effectively regulates the expression of glutamate receptor subunit genes in the brain and restores the imbalance.

Currently, significant progress has been made in the study of the pharmacological activity of 20(R)—ginsenoside [108]. Correlative studies on the pharmacological activity and stereoselective activity of 20(R)-ginsenosides will help to validate the function and value of the corresponding 20(S)-isomers. The role of 20(R)-ginsenosides in the chemotherapy and treatment of cancers is one of the most widely researched areas, and their therapeutic efficacy is well known in the clinical setting [109]. Ginsenoside Rg2 has two photometric isomers, 20(R)-Rg2 and 20(S)-Rg2.

Studies on the differences in the actions of retinol isomers are gradually becoming the focus of current research. Different structures usually have different effects, and this study compared the effects of natural ginsenoside Rg2, R-type, and S-type. The effect of the high dose of 20(R)-Rg2 was significantly better than that of the S-type. The effects of medium and high doses of natural Rg2 were significantly better than those of the S-type, and the effects of high doses of natural Rg2 on intracellular Ca^2+^ concentration, SOD, and MDA content were significantly better than those of the R-type. These results indicated that the anti-ischemia-reperfusion injury effect of 20(R)-Rg2 was superior to that of 20(S)-Rg2, but inferior to that of natural Rg2.

Ginsenoside Rg2 may play a role in preventing neuronal injury in CIRI by increasing the antioxidant capacity and calcium overloading capacity of neurons [34,35,36,37,38,39,40,41,42,43,44,45,46,47,48,49,50,51,52,53,54,55,56,57,58,59,60,61,62,63,64,65,66,67,68,69,70,71,72,73,74,75,76,77,78,79,80,81,82,83,84,85,86,87,88,89,90,91,92,93,94,95,96,97,98,99,100,101,102,103,104,105,106,107,108,109,110]. 20(R)-Rg2 was stronger than the S-type but inferior to the natural-type. Brain neuron survival was significantly decreased after OGD/R, which was prematurely inhibited by different concentrations of ginsenoside Rg2, suggesting that ginsenoside Rg2 has a neurological protective effect on this injury.

Schizophrenia (SP) is the main pathological feature of AD, and Aβ is the main component of SP. Aβ is produced by the cleavage of APP. APP is a transmembrane protein widely found in various tissues of the body. Under normal conditions, it is cleaved to produce only a very small amount of Aβ [111]. However, the metabolism of APP can be abnormal under the influence of a variety of genetic and non-genetic factors. Excess Aβ is produced and deposited in the cerebral cortex and hippocampus to form SP, which plays a very important role in the pathogenesis of AD. Aβ deposition is cytotoxic and induces neuronal apoptosis. Bcl-2 is an important inhibitor of neuronal apoptosis, whereas Bax promotes neuronal apoptosis, and the two form a balanced system. It was found that Aβ can reduce the expression of Bcl-2 and increase the expression of Bax, thus inducing neuronal apoptosis.

In addition, Aβ can lead to abnormal synaptic function, which is an important cause of cognitive impairment [112]. It was found that ginsenoside Rg2 increased the expression of Bcl-2 and HSP70 and decreased the expression of Bax and P53. Ginsenoside Rg2 and Rh1 can regulate the expression of apoptotic proteins and inhibit neuronal apoptosis induced by Aβ deposition, which is useful for the prevention of AD.

BDNF is a member of the neurotrophin growth factor family, which is involved in neuronal plasticity in several brain regions. Much evidence suggests that BDNF expression is reduced by psychological stress [113]. Therefore, lack of neurotrophic support can lead to severe depression [114]. In addition, disruption of sleep homeostatic processes leads to higher stress vulnerability and is often associated with stress-related psychiatric disorders. In clinical practice, chronic pain patients are often associated with anxiety and depression [115], and the Cardio-cerebral infarction (CCI) model is the most classical and commonly used pain model. Its pain characteristics are very similar to neuropathic pain, including spontaneous and triggered pain. However, both Rg2 and Rh1 prolonged the mechanical foot-shrinking reflex threshold and thermal foot-shrinking latency and alleviated anxiety and depression in CCI model rats. The mechanism of action of both may be through the upregulation of the BDNF signaling pathway. Disruption of synaptic plasticity is one of the pathogenic mechanisms of depression, and BDNF, as a synaptic modulator, has an important role in the treatment of depression. The hippocampus and prefrontal cortex are brain regions associated with depression. Stress and depression decrease BDNF expression and function in these two areas. People with depression also have lower levels of BDNF in their blood. Antidepressants can increase BDNF expression.

A decline in central cholinergic neurologic function leads to cognitive impairment [116]. The ginsenosides Rg2 and Rh1 can reverse the decline and improve learning and memory by increasing the expression of Ach. Ginsenosides Rg2 and Rh1 inhibit AChE activity and increase BDNF expression. In addition, long-term administration of ginsenosides Rg1 and Rh1 improved learning by increasing BDNF.

PTSD behavior is a serious psychiatric condition, hyperactivity of the hypothalamic-pituitary-adrenal (HPA) axis caused by stress is associated with the pathogenesis of depression and is accompanied by dysfunction of the hypothalamic-pituitary-gonadal (HPG) axis [117]. The HPA axis is the biological system responsible for the stress response; when stress occurs, the hypothalamus releases corticotropin and arginine vasopressin into the pituitary gland to produce corticotropin, which stimulates the adrenal glands to produce cortisol or corticosterone. Cortisol plays an important role in learning, memory, and emotion [118]. However, too much cortisol produced by stress can alter brain functions associated with depression, such as the hippocampus, prefrontal cortex, and amygdala. Dysfunction of the prefrontal cortex or hippocampus has been reported to be associated with the neuropathogenesis of PTSD [119]. And interestingly, Rg2 effectively blocked PTSD-induced depressive, anxiety-like behavior. In addition, fear-like and anxiety-like behavioral deficits in stressed animals were mitigated by Rg2 without affecting motor activity, which is consistent with the antidepressant-like effect of Rg2.

The PI3K/Akt signaling pathway plays an important role in neuroprotection and apoptosis, and activates MAPK to produce a neuroinflammatory response, leading to nerve cell death [120]. Studies have shown that ginsenoside Rh1 alleviates apoptosis and oxidative stress by activating the PI3K/Akt pathway [69].

TLR4 belongs to the family of model receptors and is the most important and common TLR [121]. TLR4 has been found to play a particularly important role in brain injury responses [122]. The MyD88 adaptor protein plays an important role in the TLR4 signaling pathway. Studies have shown that the TLR4 pathway is divided into the myeloiddifferentiationfactor88 (MyD88)-independent pathway and the MyD88-dependent pathway [123]. P65 belongs to the NF-kB family [124]; it has been found that the NF-κB/p65 signaling pathway plays an important role in chronic inflammation, and the increase in the p65 activation level is one of the pathogenesis factors of neurodegenerative diseases [125].

Factors associated with the TLR4/NF-κB signaling pathway are upregulated in the hippocampus in an animal model of pre-eclampsia. It was found that Rg2 alleviated neurological injury and blood-brain barrier damage in a pre-eclampsia animal model and attenuated the downregulation of occludin and Claudin-5 expression by modulating the TLR4/NF-κB signaling pathway and inhibiting the increase in TLR-4, MyD88, phospho-IκBα (p-IκBα), and p-NF-κB expression. Based on these findings, we suggest that ginsenosides Rg2 and Rh1 may be effective functional compounds for the treatment of neurological-related diseases.

In conclusion, the pathogenesis of neurological diseases is very complex. Therefore, it is urgent to find fast-acting, low-toxicity, and safe herbal medicines to fight against these diseases. Rg2 and Rh1 belong to the ginsenotriol type of saponins. Rg2 and Rh1 are ginsenotriol saponins and have been shown to exert pharmacological activities by regulating various signaling pathways.

## 5. Conclusions

The innovation of this study is to update the research related to the ginsenoside monomer components Rg2 and Rh1 in protecting humans encountering neurological diseases. As a natural resource of medicine and food, the exploitation of ginseng has also been of great interest. Ginsenosides, the active ingredients of ginseng, are natural biomacromolecular compounds, which are mainly known for their protective effects on the nervous and cardiovascular systems, as well as immunomodulatory, anti-tumor, and antioxidant effects. Agents of natural origin usually arouse great interest, and the current discovery of new ginseng components is still in progress. With the continuous development and advancement of science and technology, the research on ginseng is also deepening. As the main active ingredients of ginseng, ginsenosides Rg2 and Rh1 have very wide clinical applications in the treatment of neurological diseases. Studies have shown that ginsenosides and their derivatives have great medicinal potential in the prevention and treatment of different diseases, but no report has been made on the co-administration of Rg2 and Rh1 or the combination with the traditional clinical drugs for the treatment of neurological diseases; and with the increasing incidence of neurological diseases in the social groups, it can be seen that this is also a new angle for the development of effective preventive and therapeutic drugs with natural active ingredients. At the same time, we firmly believe that the Chinese medicine ginseng will open up new horizons for the pharmaceutical industry in the future.

## Data Availability

The data presented in this study are available on request from the corresponding author. The data are not publicly available due to data sharing is not applicable to this article.

## Abbreviations

PD	parkinson’s disease
DA	dopamine
6-OHDA	6-Hydroxydopamine hydrobromide
ERK	extracellular signal-regulated protein kinase
JNK	c-jun N-terminal kinase
NB	neuroblastoma
MDA	malondialdehyde
NO	nitric oxide
Glu	glutamic acid
NMDA	*N*-Methyl-d-aspartic acid
APP	amyloid Precursor Protein
Aβ1-40	amyloid β-Protein (1-40)
Aβ25-35	amyloid β-Protein (25-35)
Bcl-2	B-cell lymphoma-2
Bax	Bcl-2-associated X
AChE	Acetylcholinesterase
ChAT	Choline acetyltransferase
ACh	Acetylcholine
SY	synaptophysin
ICH	intracerebral hemorrhage
TLR4	toll-like Receptor 4
NF-κB	nuclear factor kappa-B
SD	Sleep deprivation
HPA	hypothalamic-pituitary-adrenal
HPG	hypothalamic-pituitary-gonadal
MyD88	myeloiddifferentiationfactor88
PI3K	Phosphatidylinositide 3-kinases
Akt	Protein kinase B
VD	Vascular dementia
CICR	cerebral ischemia-reperfusion injury
HSP70	heat shock protein 70
P53	recombinant Tumor Protein
OGD/R	Oxygen glucose stripping/Reperfusion
CCI	chronic constric-tion injury
BDNF	brain-derived neurotrophic factor
TrkB	Tyrosine Kinase receptor B
shRNA	short hairpin RNA
PTSD	Post-traumatic stress disorder
SPS	single-long stress
EPMT	elevated plus maze test
5-HT	5-hydroxytryptamine
5-HIAA	5-hydroxyindoleacetic acid
CRH	corticotropin releasing hormone
Cort	corticosterone
ACTH	adrenocorticotropic hormone
AD	Alzheimer’s disease
CNS	Central Nervous System
CN	Calcineurin
NFAT	Nuclear factor activating T cells
SP	Schizophrenia
CCI	Cardio-cerebral infarction
p-IκBα	phospho-IκBα
